# Virtual Screening of Bacteriocins From Lactic Acid Bacteria Against Monkeypox DNA Polymerase: Sakacin P and Mundticin KS Emerge as Promising Candidates

**DOI:** 10.1002/fsn3.72219

**Published:** 2026-08-06

**Authors:** Melisa Z. Karaman, Karan Goel, Fernando Berton Zanchi, Aykut Ozdarendeli, Somdutt Mujwar, Ahmet E. Yetiman, Ozkan Fidan

**Affiliations:** ^1^ Department of Bioengineering, Faculty of Life and Natural Sciences Abdullah Gul University Kayseri Türkiye; ^2^ Chitkara College of Pharmacy Chitkara University Rajpura Punjab India; ^3^ Bioinformatic and Medicinal Chemistry Laboratory Fiocruz/RO Porto Velho Rondônia Brazil; ^4^ Department of Microbiology, Faculty of Medicine Erciyes University Kayseri Kayseri Türkiye; ^5^ Vaccine Research, Development and Application Centre (ERAGEM) Erciyes University Kayseri Türkiye; ^6^ Department of Food Engineering, Faculty of Engineering Erciyes University Kayseri Türkiye

**Keywords:** bacteriocins, molecular docking, molecular dynamics simulation, monkeypox virus, Mundticin KS, Sakacin P

## Abstract

The monkeypox virus (MPXV) has recently risen to be a significant global health threat and currently there are no approved antiviral agents, which makes it necessary to develop new antiviral strategies. In this study, we employed in silico techniques to investigate whether bacteriocins could be potent inhibitors of the MPXV DNA polymerase (MPDP). At first, the MPXV DNA polymerase enzyme chain was extracted from the Cryo‐EM structure of MPXV DNA replication complex and was then assessed using SWISS‐MODEL/QMEAN for structural quality. The structures of selected bacteriocins were either retrieved from the Protein Data Bank or predicted using AlphaFold. The quality of these predicted models was measured by LGscore. Moreover, the physicochemical properties of the selected bacteriocins, such as Sakacin P and Mundticin KS, were analyzed to assess their molecular stability and compatibility with the molecular docking process. The results of protein‐peptide docking simulations on the HADDOCK platform showed that among all the bacteriocins tested, Sakacin P and Mundticin KS had the highest binding affinities toward MPXV DNA polymerase. The analysis of the docking experiments also revealed vital stabilizing interactions, such as the formation of hydrogen bonds, ionic linkages, and π–π stacking, which were crucial in the strength of the protein–ligand complexes. Subsequently, the molecular dynamics (MD) simulations further confirmed the stability of the protein–bacteriocin complexes. Our results indicate that bacteriocins, especially Sakacin P and Mundticin KS, can be considered potential antiviral agents against MPXV by interfering with its DNA replication mechanism. The present work shall serve as a reference for future laboratory testing and for the promising innovation of bacteriocin‐based drugs targeting MPXV.

## Graphical abstract

This study employs an in silico virtual screening workflow to evaluate lactic acid bacteria (LAB)‐derived bacteriocins as potential inhibitors of the monkeypox virus DNA polymerase (MPDP). Protein‐peptide docking simulations using the HADDOCK platform revealed that Sakacin P and Mundticin KS possess the highest binding affinities for the MPDP target, stabilized by key molecular interactions such as hydrogen bonds, ionic linkages, and π–π stacking. Furthermore, comprehensive molecular dynamics (MD) trajectory analyses confirmed the structural stability and compactness of both Sakacin P–MPDP and Mundticin KS–MPDP complexes, highlighting these bacteriocins as promising candidates for future anti‐MPXV drug development.

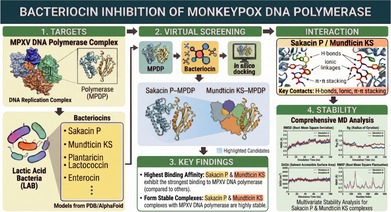

## 1 Introduction

Monkeypox virus (MPXV) was first discovered in 1958 when an unidentified disease appeared in monkeys in Africa (Luo et al. 2024). Scientists discovered a new virus in the infected monkeys. Because the monkeys had similar blisters on the skin as those seen in smallpox, the name of the disease was changed to “monkeypox” (Luo et al. 2024; Sun et al. 2024). On 14 August 2024, the WHO declared the ongoing MPXV outbreak a “Public Health Emergency of International Concern” (PHEIC), which is the second MPXV‐related PHEIC within 2 years. The Africa Centres for Disease Control and Prevention (Africa CDC) also declared a regional emergency on 13 August 2024, following the report of 35,341 MPXV cases and 840 deaths across 13 African countries between January 1 and September 23, 2024 (Zumla et al. 2024).

MPXV is an enveloped double‐stranded DNA virus, which belongs to the genus *Orthopoxvirus* of the family Poxviridae (Luo et al. 2024; Kameli et al. 2025). *Orthopoxvirus* also includes Cowpox, Smallpox, and Vaccinia viruses (Derhab 2024). The clinical signs of this viral disease are similar to those of smallpox, but the severity might mostly change from one patient to another or among conditions (Islam et al. 2023). As a zoonotic virus, MPXV is capable of spreading from animals to humans as well as among humans. In general, human‐to‐human transmission takes place when a healthy person makes close contact with an infected one. The virus then gains entry into the new host through respiratory droplets, sexual interaction, or direct contact with infectious skin lesions and bodily fluids. The disease has a reported fatality rate ranging from 1% to 8% (Farasani 2022; Lum et al. 2022; Letafati and Sakhavarz 2023). Nevertheless, there is not an exact treatment or vaccine for MPXV infection. This may be possible through cross‐immunity when patients get a smallpox vaccination. Previous years, MPXV prevalence has increased substantially. This rise may be attributed to decreased herd immunity to smallpox as a result of the cessation of regular vaccination after its global eradication. Given that the smallpox vaccine was reported to be about 85% effective in preventing MPXV, post‐exposure vaccination can help reduce disease severity or potentially halt its progression (Kameli et al. 2025). Moreover, several antiviral drugs, such as tecovirimat, cidofovir, and brincindofovir, can be employed in the treatment of MPXV infections, but their effectiveness has not been fully affirmed (Sun et al. 2024; Kameli et al. 2025). Consequently, there remains an urgent demand for novel and effective therapeutic options against MPXV.

Lactic acid bacteria (LAB) can produce different low molecular weight chemicals, including hydrogen peroxide, carbon dioxide, diacetyl, organic acids, ethanol, and bacteriocins, which can suppress the growth of competing microorganisms (Siedler et al. 2019; Vieco‐Saiz et al. 2019; Amenu et al. 2025). Bacteriocins are ribosomally synthesized antimicrobial peptides capable of inhibiting or killing closely related bacterial species. However, the producing strains remain unaffected by their own bacteriocins due to the presence of specific immunity proteins (Yang et al. 2014; Lahiri et al. 2022; Niamah et al. 2024). Bacteriocins have diverse biotechnological uses, ranging from their application as natural preservatives in the food and dairy sectors to clinical roles as potential substitutes for certain antibiotics. In addition, they exhibit medical relevance due to their anticancer, antiviral, and antiprotozoal properties (Soltani et al. 2021; Cheruvari and Kammara 2025).

Several studies have revealed bacteriocins possess antiviral activity against a wide range of viruses (Cheruvari and Kammara 2025). Enterocin CRL35, produced by *Enterococcus faecium* CRL35, was the very first bacteriocin reported to have antiviral properties. This peptide was able to exert inhibitory effects on both Herpes simplex virus type 1 and type 2 (HSV‐1 and HSV‐2), which are responsible for serious health complications such as corneal blindness, genital ulcerations, and encephalitis (Wachsman et al. 1999, 2003). Similarly, Enterocin AAR‐71 was highly effective against the Coliphage HSA virus, as it completely prevented the formation of viral progeny (Qureshi et al. 2006; Dicks and Grobbelaar 2021). In addition, Staphylococcin 188, produced by *Staphylococcus aureus* AB188 and therefore not derived from lactic acid bacteria, revealed antiviral properties against Influenza virus, Newcastle disease virus, and Coliphage HAS (Saeed et al. 2006; Reuben and Torres 2024). Moreover, Labyrinthopeptin A from *Actinomadura namibiensis* DSM 6313 was another bacteriocin with HSV‐1 and HIV‐1 antiviral properties (Férir et al. 2013; Ramírez‐Rendón et al. 2023). In addition, *Lactobacillus delbrueckii* subsp. *bulgaricus* 1043 is known to produce a nontoxic bacteriocin with virucidal activity against the Influenza virus (Serkedjieva et al. 2000). Furthermore, duramycin was shown to bind to phosphatidylethanolamine in the envelope of the Zika virus, thereby blocking the TIM1 receptor and reducing infection in placental cells and explants. Similarly, micrococcin P1 has been reported to inhibit entry of the Hepatitis C virus without affecting the secretion of viral particles (Elalem et al. 2021).

To our knowledge, the use of bacteriocins as potential drugs for MPXV has not been explored in the literature yet. Hence, an in silico study was directed to evaluate bacteriocins of lactic acid bacteria against the DNA polymerase of MPXV (MPDP). A virtual screening of 19 bacteriocins against DNA polymerase was carried out by protein–protein docking and molecular dynamics simulation. Our in silico analysis revealed that Sakacin P and Mundticin KS are the most promising bacteriocin candidates with strong binding affinity and potent physicochemical characteristics as DNA polymerase inhibitors for MPXV.

## 2 Materials and Methods

### 2.1 In Silico Preparation and Confirmation of Bacteriocins and Receptors

Candidate bacteriocins from lactic acid bacteria were selected based on the availability of complete peptide sequences and suitable structural information for docking, either as experimentally determined structures in the RCSB PDB (https://www.rcsb.org/) or as AlphaFold‐predicted models available through UniProt‐linked (https://www.uniprot.org/) resources (Erol et al. 2021). Nineteen bacteriocins and two antiviral peptides (controls) met these inclusion criteria and were carried forward for screening. Control peptide structures were used as controls, obtained from the Comprehensive Database of Antiviral Peptides and Proteins (DRAVP) (Liu et al. 2023).

For AlphaFold‐predicted models, peptides with an average per‐residue confidence score (pLDDT) of 50 or higher were considered for inclusion. It is important to note that AlphaFold‐derived peptide models with mean pLDDT values above 50 were retained for preliminary screening only, because pLDDT is a residue‐level confidence metric and lower average pLDDT in short peptides may reflect conformational flexibility/disorder rather than uniformly poor local geometry; therefore, final model selection for docking relied on orthogonal validation criteria, including Ramachandran stereochemical assessment and ProQ LGscore evaluation (Laskowski et al. 1993; Wallner and Elofsson 2003; Jumper et al. 2021; McDonald et al. 2023; Terwilliger et al. 2023). The average pLDDT values for Uniprot sourced bacteriocins were acquired from AlphaFold Protein Structure Database (https://alphafold.ebi.ac.uk/). Additionally, the structure predictions were performed for Mundticin_KS (PDB: 2ZRR), Mellitin (DRAVPe00837), and UL54 (DRAVPe01393) using the AlphaFold Server (https://alphafoldserver.com). The average pLDDT values for these structures were calculated in PyMOL v3.1.0 (https://github.com/schrodinger/pymol‐open‐source; Schrodinger LLC, New York, NY) using CIF files obtained from the AlphaFold Server. Pure 3D structures generated through X‐ray diffraction or solution NMR were retrieved from the RCSB Protein Data Bank for the available bacteriocins. For the bacteriocins with NMR‐derived structures, the first model of the NMR ensemble with the lowest energy was selected.

Cryo‐EM structure of monkeypox virus DNA replication holoenzyme (F8, A22, and E4 complex) without DNA at 2.76 Å (PDB ID: 8HOY) was also downloaded from the Protein Data Bank (PDB) (Xu et al. 2023). 8HOY was selected as it provides a high‐resolution structure with a more complete representation of the DNA polymerase among the available deposits in PDB at the time of our analysis (2024). The monkeypox virus DNA polymerase enzyme chain was extracted from the complex using PyMol v3.1.0. The extracted chain was evaluated using SWISS‐MODEL to assess structure quality based on GMQE (Global Model Quality Estimate) and QMEANDisCo global scores (Waterhouse et al. 2018; Studer et al. 2020). All solvent molecules found in the PDB files were removed using BIOVIA Discovery Studio 2024 Client (Dassault Systèmes, Vélizy‐Villacoublay, France). Structural validation of all protein and peptide models was performed using the PROCHECK tool via the SAVES v6.1 server (https://saves.mbi.ucla.edu/). Moreover, the PROCHECK tool also generated Ramachandran scores, which represent the percentage of amino acids found in the most favored regions. Additionally, the structural quality of the all peptide and protein structures was assessed, and authentication was carried out using the ProQ server (https://proq.bioinfo.se/cgi‐bin/ProQ/ProQ.cgi).

### 2.2 Protein–Protein Docking and Physicochemical Features

HADDOCK v2.4 was employed in protein–protein docking simulations (Honorato et al. 2024). Active residues from the positively charged groove of the F8 thumb domain of MPXV DNA polymerase (MPDP), responsible for DNA binding, were acquired from the literature. Reported active residues comprised of ARG302, LYS308, LYS340, LYS525, ARG674, LYS803, LYS804, LYS805, ARG832, ARG833, LYS973, ARG974, and ARG1000, located within the F8 thumb domain of the MPDP (Xu et al. 2023). The server with the default protocol and parameter sets was utilized for docking and refinement. Passive residues were defined automatically in proximity to the active residues. The top‐ranking complexes from HADDOCK were downloaded and submitted to the PROtein binDIng enerGY prediction (PRODIGY) server (Jiménez‐García et al. 2019) to calculate binding energies at the temperature of 310 K (36.85°C). Subsequently, protein–protein residue interactions were determined through the Residue Interaction Network Generator (RING, v4) web server using the PDB dataset acquired from PRODIGY. Closest nodes, strict threshold, add hydrogens, and include water parameters were chosen for residue interaction analysis in the RING. All figures were generated using PyMol. In the article, we defined the MPDP as chain A and the bacteriocins as chain B. For example, A805‐B23 denotes the interaction between the 805th amino acid of the MPDP and the 23rd amino acid of the bacteriocin. The physicochemical characteristics of bacteriocins, including amino acid sequences and length, molecular weight, isoelectric point (pI), instability index, aliphatic index, grand average of hydropathicity (GRAVY) score, and estimated in vitro half‐life in mammalian reticulocytes, were predicted using Expasy's ProtParam tool (https://web.expasy.org/protparam/) to confirm their stability.

### 2.3 Molecular Dynamics Simulation

First, to assess the stability and dynamic behavior of MPDP when exposed to Sakacin P and Mundticin KS, two systems for each bacteriocin were modeled: one in the apo form and the other complexed with the bacteriocin. GROMACS 2024.2 was used to carry out the molecular dynamics (MD) simulation with the Visual Dynamics interface for script generation, and the AMBER99 force field was utilized (Wang et al. 2004; Abraham et al. 2015; Vieira et al. 2023). Electrostatic interactions were calculated using the particle mesh Ewald (PME) method, with a 12 Å cutoff. Each system was introduced into a cubic periodic box which was automatically set to extend 1 nm from the outermost protein atoms in all directions and was then solvated with TIP3P water molecules (Hardy et al. 2015; Briand et al. 2025). The systems were neutralized by the addition of NaCl to bring the concentration to 0.15 M. Energy minimization was achieved by two steps in 2000 steps of steepest descent and then 2000 steps of conjugate gradient, or until the maximum force was < 1000 kJ mol^−1^ nm^−1^. Initial velocities were determined based on a Maxwell‐Boltzmann distribution at 300 K.

Equilibration was performed in two stages using NVT and NPT ensembles for 200 ps each, respectively temperature coupled by the V‐rescale thermostat and pressure coupled by the Parrinello‐Rahman barostat. Production runs were carried out at 300 K and 1 atm without restraints, using isotropic coupling. All covalent bonds involving hydrogen atoms were fixed by the LINCS algorithm (Araki et al. 2021; Bullerjahn et al. 2023). A 2 fs integration time step was applied, and coordinates were saved every 10 ps. Lastly, 500 ns simulations were performed for both the apo system and the bacteriocin‐bound complex configurations. Simulation trajectories were inspected with standard tools from the GROMACS package (Balijepalli et al. 2013). Root mean square deviation (RMSD) and root mean square fluctuation (RMSF) were determined for each system based on all protein atoms, and the starting structure from the production run was used as a reference. Receptor–ligand hydrogen bonds (H‐bonds) were measured during the whole time of the simulations. The radius of gyration (Rg), which indicates the overall degree of compactness, and the solvent‐accessible surface area (SASA), which is the surface of the molecule exposed to solvent molecules, were calculated following the protocols described in the GROMACS manual. Receptor–ligand binding interactions were additionally assessed by Molecular Mechanics Poisson‐Boltzmann Surface Area (MM‐PBSA) calculations with the help of the gmx_MMPBSA package (Genheden and Ryde 2015; Paissoni et al. 2015; Akkus et al. 2023). The last 100 ns of each trajectory were taken into account for these analyses. Both enthalpic and entropic contributions to binding were considered in the method and the Gibbs free binding energy was estimated as Δ*G*_bind_ = Δ*H* − *T*Δ*S*, with *T* being the temperature of the simulation (300 K). All MD simulation setup files including MDP files, topology/parameter files, and trajectory files were uploaded to Zenodo (https://doi.org/10.5281/zenodo.19671741).

## 3 Results

### 3.1 Prediction and Verification of Bacteriocins and Monkeypox DNA Polymerase

**FIGURE 1:**
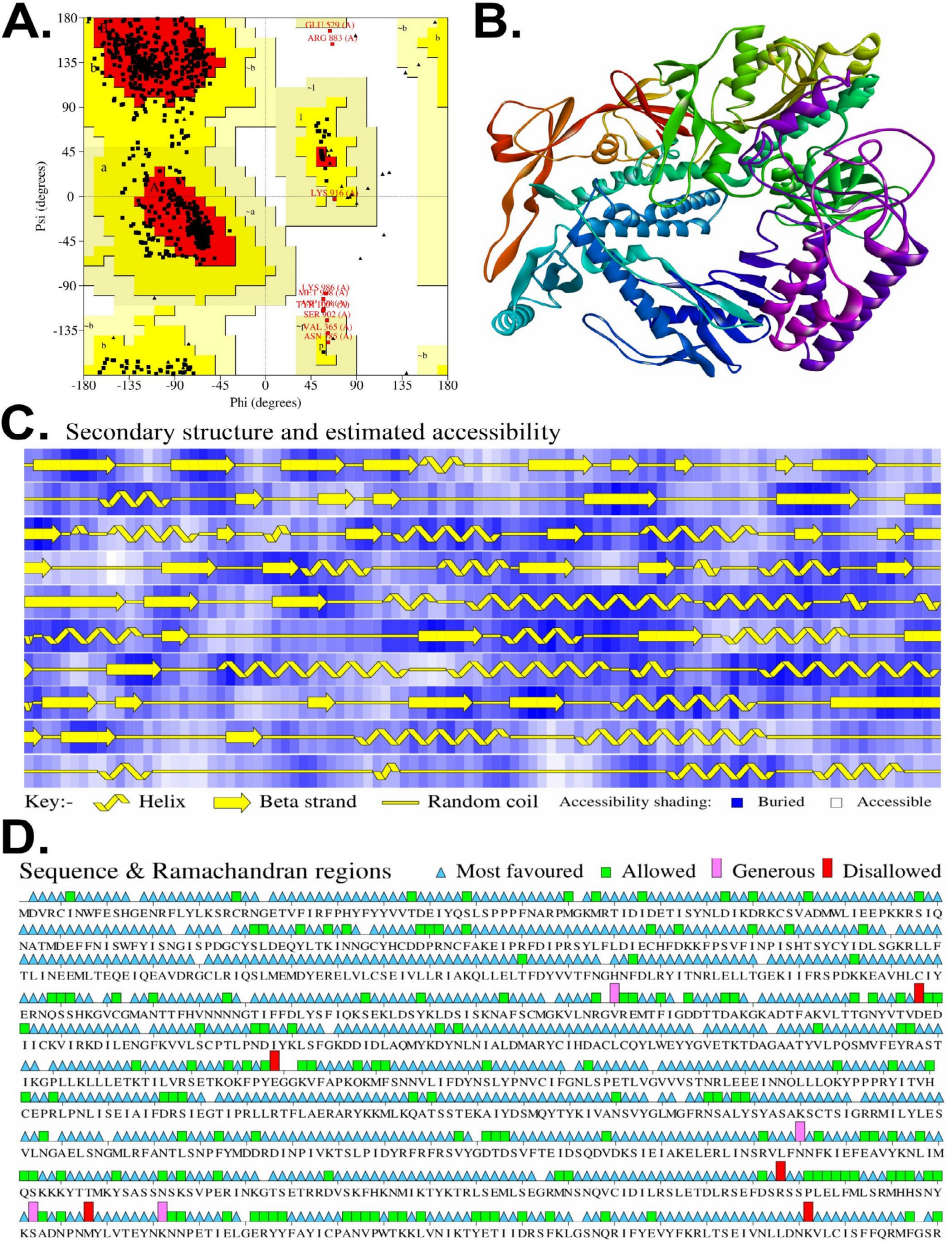
Structure confirmation of the MPXV DNA polymerase. (A) Confirmation of the 3D structure predicted via the Ramachandran plot. (B) Cartoon illustration of the 3D structure of MPXV DNA polymerase. (C) The secondary structure of the MPXV DNA polymerase comprises α‐helices, β‐sheets, and random‐coiled polypeptide configurations. (D) The peptide sequence and Ramachandran fields are depicted.

Determining the 3D structures of MPXV DNA polymerase (receptor) and bacteriocins (ligands) from their amino acid sequences was an essential step in assessing receptor–ligand binding affinities. The selection of a protein modeling method based on amino acid sequences relies on the resemblance to established templates in the database. Homology modeling is favored when the similarity exceeds 30%. The interpretation of computationally generated data is straightforward and dependable, effectively bridging the gap between the template and the unknown protein structure (Waterhouse et al. 2018). The structure of MPDP was derived from human monkeypox viral replication complexes (PDB ID: 8HOY) in the PDB database. The removal of subunits from protein complexes can destabilize the remaining components or cause them to adopt different conformations (Levy et al. 2008; Marsh and Teichmann 2015). Thus, structure validation of the MPDP was conducted utilizing the PROCHECK tool through the SAVES v6.1 structure validation server (Figure 1). For the MPDP, Ramachandran plot statistics revealed that 83.1% of residues lie in the most favored regions, 15.8% in additional allowed regions, 0.4% in generously allowed regions, and 0.6% in disallowed regions. The Ramachandran plot serves as a universal criterion for validating predicted protein structures by estimating their stability (Kumari et al. 2023). The secondary structure elements (helix, beta strand, random coil) and estimated accessibility (buried, accessible) were annotated. This provided a visual representation of the protein's fold and surface properties. Figure 1 also illustrates the distribution of specific amino acids in the polypeptide chain across their respective Ramachandran regions: most favored, allowed, generous, and disallowed. In addition, the structural models for MPDP were generated using SWISS‐MODEL and subsequently evaluated for global and local structural quality using Qualitative Model Energy Analysis (QMEAN) (Benkert et al. 2011). QMEANDisCo predicts interatomic distance constraints by incorporating information derived from experimentally validated homologous protein structures, which are used as templates to assess the reliability of the modeled structure (Studer et al. 2020). The QMEANDisCo and GMQE values, which range from 0 to 1, were considered acceptable indicators of model quality. For MPDP, both scores were 0.86, supporting the reliability of the predicted structure.

The bacteriocins and control antiviral peptides included in this study were likewise subjected to structural prediction and validation prior to docking. Bacteriocin models were generated using AlphaFold‐based resources, and all structures were subsequently examined by PROCHECK and ProQ. AlphaFold can directly predict raw atom coordinates with a diffusion module due to its capacity to work on amino acid‐specific frames and side chain torsion angles (Abramson et al. 2024). More than 90% of the amino acid residues in the bacteriocin models were located inside the favorable region of the Ramachandran plot. Moreover, the quality of these structures was evaluated using the ProQ server to determine the Levitt‐Gerstein (LG) score. The LG score measures similarity to a known structure by superimposing two structures and was utilized to assess the quality of these structures. Protein/peptide models exhibiting an LG score > 4 were deemed of high quality (Levitt and Gerstein 1998). All models had LG scores between 6.605 and 11.927. These results confirmed that the peptide structures used for docking possessed acceptable stereochemical and overall structural quality.

To strengthen the comparative framework of the screening, two experimentally validated antiviral peptides were included as controls: Melittin (DRAVPe00837) and UL54 (1221–1242) (DRAVPe01393). Melittin is a 26‐amino‐acid peptide derived from the venom of *Apis mellifera* and is recognized as a broad‐spectrum antiviral peptide with reported activity against several enveloped and non‐enveloped viruses, including Herpes simplex virus, Junin virus, İnfluenza A virus, Respiratory syncytial virus, Vesicular stomatitis virus, Enterovirus A71, and HIV (Albiol Matanic and Castilla 2004; Memariani et al. 2019; Liu et al. 2023). UL54 (1221–1242), in contrast, is a 22‐amino‐acid synthetic peptide derived from the C‐terminal region of the human cytomegalovirus polymerase subunit UL54 and has been experimentally shown to inhibit the UL54–UL44 interaction required for viral DNA polymerase activity, making it a mechanistically relevant polymerase‐targeting antiviral control (Loregian et al. 2003; Liu et al. 2023). Importantly, both control peptides also showed excellent structural quality in the present study, with average pLDDT values of 92.21 for Melittin and 85.49 for UL54 (1221–1242), LG scores of 11.713 and 10.107, respectively, and 100% of residues located in favored/allowed Ramachandran plot regions. These results confirmed that the control peptides were not only biologically relevant, but also structurally robust references for benchmarking the bacteriocin‐based screening against MPDP.

### 3.2 Molecular Docking and Physicochemical Properties Predictions

The interactions between computer‐assisted MPDP and bacteriocins were analyzed to elucidate their binding affinities through molecular docking techniques. The molecular docking, or binding pose, is determined by multiple three‐dimensional interactions, including hydrogen bonding, hydrophobic interactions, and other non‐covalent interactions that occur within the DNA‐binding groove of MPDP during complex formation with the bacteriocins (Del Conte et al. 2024). This approach enables the virtual screening of inhibitory molecules for target proteins, while effective docking tools can evaluate and rank the candidate ligands using a scoring system that reflect the strength and nature of their predicted interactions (Kumari et al. 2023). For this purpose, this study employed HADDOCK and PRODIGY to estimate binding energies, while the RING web server was utilized to identify residue interactions. The complete docking results are presented in Table 1. Among the 19 bacteriocins screened, Sakacin P from *Latilactobacillus sakei* and Mundticin KS from *Enterococcus mundtii* emerged as the most promising MPDP inhibitor candidates, with a predicted binding energy of −16.7 and −17.0 kcal/mol and an estimated dissociation constant of 1.6 × 10^−12^ and 9.6 × 10^−13^ M, respectively. Plantaricin W beta also showed strong predicted binding (−16.2 kcal/mol), yet its overall molecular characteristics remained inferior to those of Sakacin P and Mundticin KS. Therefore, Sakacin P and Mundticin KS were shortlisted as the most compelling peptide candidates for subsequent investigation against MPDP.

**TABLE 1:** Structure confirmation parameters and binding energies of bacteriocins versus monkeypox DNA polymerase.

Origin	Bacteriocin/Peptide	Average pLDDT^a^	LG score^b^	Ramachandran score (%)^c^	Binding energy (kcal/mol)	*K*_d_ (M) at 36.85°C
*Enterococcus mundtii*	Mundticin_KS	79.92 (High)	7.838	98.8	−17	9.6e‐13
*Latilactobacillus sakei*	Sakacin_A	55 (Low)	11.168	100	−13	6.7e‐10
*Latilactobacillus sakei*	Sakacin_P	75.88 (High)	9.050	100	−16.7	1.6e‐12
*Lactiplantibacillus plantarum*	Plantaricin_W_beta	52.78 (Low)	9.456	98.2	−16.2	4.00e‐12
*Lactiplantibacillus plantarum*	Plantaricin_W_alpha	60.06 (Low)	11.292	100	−12.3	2.3e‐09
*Pediococcus acidilactici*	Pediocin_PA1	76.31 (High)	11.071	98	−12.1	2.8e‐09
*Lactococcus lactis* subsp. *lactis*	Nisin	58.44 (Low)	11.553	100	−12.2	2.3e‐09
*Leuconostoc mesenteroides*	LeucocinC_TA33a	63.72 (Low)	10.580	100	−13.5	3.2e‐10
*Leuconostoc mesenteroides*	LeucocinB_TA33a	72.56 (High)	10.533	95.4	−11.1	1.5e‐08
*Leuconostoc mesenteroides*	LeucocinB_TA11A	55.66 (Low)	10.445	96	−13.7	2.1e‐10
*Lactococcus lactis* subsp. *lactis*	Lactococcin_G_beta	72.62 (High)	11.090	100	−10.2	6.1e‐08
*Lactococcus lactis* subsp. *lactis*	Lactococcin_G_Alpha	82.06 (High)	10.781	100	−12.7	1.1e‐09
*Lactococcus lactis* subsp. *lactis*	Lactococcin_Mmfii	64.75 (Low)	11.062	96.8	−9.7	1.4e‐07
*Lactococcus lactis* subsp. *lactis*	Lacticin_481	56.72 (Low)	11.927	97.8	−11.3	1.1e‐08
*Enterococcus faecium*	Enterocin CRL_35	71.94 (High)	6.605	100	−13.7	2.1e‐10
*Enterococcus faecalis*	Enterocin_EJ97	76.06 (High)	11.448	100	−13.5	3.2e‐10
*Enterococcus faecalis*	Enterocin_A	68.56 (Low)	11.154	100	−11.5	7.8e‐09
*Carnobacterium maltaromaticum*	Carnobacteriocin B2	58.59 (Low)	11.102	100	−14.8	3.5e‐11
*Lactobacillus acidophilus*	AcidocinJ_1132beta	77.5 (High)	11.031	100	−10.8	2.5e‐08
*Apis mellifera*	Mellitin (DRAVPe00837)^e^	92.21 (Very high)	11.713	100	−10.9	1.9e‐08
Synthetic construct^d^	UL54 (DRAVPe01393)^e^	85.49 (High)	10.107	100	−10.8	2.5e‐08

^a^ The predicted local distance difference test (pLDDT) provides confidence in the local structure by estimating how closely the prediction matches an experimental structure. It is derived from the local distance difference test Cα (lDDT‐Cα), a score that evaluates the accuracy of local distances without requiring superposition (Mariani et al. 2013).

^b^ According to ProQ interpretation; LGscore > 1.5 fairly good model, LGscore > 2.5 very good model, LGscore > 4 extremely good model.

^c^ The percentage represents the residues in the most favored [A, B, L] and allowed [a, b, l, p] regions.

^d^ Derived from human cytomegalovirus (HCMV) UL54 protein.

^e^ The well‐known antiviral peptides served as controls. Mellitin (DRAVPe00837) is recognized as a broad‐spectrum antiviral peptide, whereas UL54 (DRAVPe01393) inhibits DNA polymerase in human cytomegalovirus (HCMV), according to the DRAVP database.

The inclusion of Melittin and UL54 (1221–1242) provided an informative antiviral control framework for contextualizing the docking results. Melittin was selected as a broad‐spectrum antiviral reference peptide due to its experimentally documented activity against diverse viral species (Albiol Matanic and Castilla 2004; Memariani et al. 2019; Liu et al. 2023; Nawaz et al. 2026). UL54 (1221–1242) was included as a mechanistically relevant comparator because it originates from a viral polymerase and has been shown to disrupt a protein–protein interaction essential for Human cytomegalovirus DNA polymerase activity (Loregian et al. 2003). Despite this established antiviral relevance, both control peptides displayed substantially weaker predicted affinity toward MPDP than Sakacin P and Mundticin KS. Melittin showed a binding energy of −10.9 kcal/mol, whereas UL54 (1221–1242) yielded a binding energy of −10.8 kcal/mol. These values were clearly less favorable than those observed for Sakacin P, Mundticin KS, and Plantaricin W beta. This comparison is particularly important because it indicates that previously reported antiviral activity, or even prior association with viral polymerase inhibition in another system, does not necessarily translate into optimal binding to MPXV DNA polymerase. In this regard, Mundticin KS and Sakacin P outperformed two biologically meaningful antiviral control peptides within the specific context of the monkeypox polymerase target.

Evaluation of physicochemical properties further reinforced the prioritization of Sakacin P and Mundticin KS (Table 2). Sakacin P had a molecular weight of 6385.13 Da, a theoretical pI of 7.81, an instability index of 21.43, an aliphatic index of 64.10, and a GRAVY score of −0.307. This combination indicates a comparatively stable peptide with a balanced hydrophilic‐hydrophobic profile, features that may favor productive interaction with the MPDP surface under physiological conditions. By comparison, Mundticin KS, despite its marginally stronger docking score, had a substantially larger molecular weight (11,101.83 Da) and an instability index of 44.96, exceeding the accepted stability threshold and reducing its suitability as a lead scaffold. Similarly, Plantaricin W beta exhibited a high instability index (47.23), suggesting lower intrinsic stability despite favorable docking performance.

**TABLE 2:** Physicochemical features of bacteriocins evaluated against monkeypox virus DNA polymerase which predicted via Expasy's ProtParam tool.

Nr.	Bacteriocin	UniProtID/PDB/DRAVP ID	Sequence	AA length	Molecular weight	pI	Instability index	Aliphatic index	GRAVY score	Estimated half‐life^a^
1	AcidocinJ_1132beta	Q9R499	GNPKVAHCASQIGRSTAWGAVSGA	24	2325.59	9.51	12.71	61.25	−0.058	30 h
2	Carnobacteriocin B2	P38580	MNSVKELNVKEMKQLHGGVNYGNGVSCSKTKCSVN WGQAFQERYTAGINSFVSGVASGAGSIGRRP	66	6993.88	9.70	19.32	60.45	−0.417	30 h
3	Enterocin_A	E5LCG3	TVDWAKATTCIAGMSIGGFLGGAFPGK	27	2657.10	7.87	24.76	68.89	0.559	7.2 h
4	Enterocin_EJ97	Q8KMU4	MLAKIKAMIKKFPNPYTLAAKLTTYEINWYKQQYGRYPWERPVA	44	5322.32	9.92	19.46	71.14	−0.589	30 h
5	EnterocinCRL_35	Q6TGQ5	MSNLKWFSGGDDRRKKAEVIITELLDDLEIDLGNESLRKVLGSYL EKLKNEGTSVPLVLSRMNIEISNAIKKDGVSLNENQSKKLKELISISNIRYGY	98	11084.74	7.91	41.34	108.37	−0.448	30 h
6	Lacticin_481	P36499	MKEQNSFNLLQEVTESELDLILGAKGGSGVIHTISHECNMNSWQFVFTCCS	51	5677.41	4.68	44.36	80.20	−0.049	30 h
7	Lactococcin_G_Alpha	P36961	GTWDDIGQGIGRVAYWVGKAMGNMSDVNQASRINRKKKH	39	4345.93	10.16	4.44	60.00	−0.841	30 h
8	Lactococcin_Mmfii	P83002	TSYGNGVHCNKSKCWIDVSELETYKAGTVSNPKDILW	37	4144.64	6.43	27.62	68.38	−0.535	7.2 h
9	Lactococcin_G_beta	P36962	KKWGWLAWVDPAYEFIKGFGKGAIKEGNKDKWKNI	35	4109.79	9.70	10.35	61.43	−0.806	1.3 h
10	LeucocinB_TA11A	Q53446	MNNMKSADNYQQLDNNALEQVVGGKYYGNGVH CTKSGCSVNWGEAFSAGVHRLANGGNGFW	61	6568.18	6.69	5.95	51.15	−0.597	30 h
11	LeucocinB_TA33a	P81052	KGKGFWSWASKATSWLTGPQQPGSPLLKKHR	31	3466.01	11.39	27.93	44.19	−0.971	1.3 h
12	LeucocinC_TA33a	P81052	KNYGNGVHCTKKGCSVDWGYAWTNIANNSVMNGLTGGNAGWHN	43	4598.04	8.79	18.09	45.35	−0.665	1.3 h
13	Mundticin_KS	2ZRR (PDB)	MSNLKWFSGGDDRRKKAEVIITELLDDLEIDLGNESLRKVLGSYLK KLKNEGTSVPLVLSRMNIEISNAIKKDGVSLNENQSKKLKELMSISNIRYGY	98	11101.83	9.19	44.96	104.39	−0.479	30 h
14	Nisin	P13068	MSTKDFNLDLVSVSKKDSGASPRITSISLC TPGCKTGALMGCNMKTATCHCSIHVSK	57	5962.98	8.99	35.11	68.42	−0.005	30 h
15	Pediocin_PA1	P29430	MKKIEKLTEKEMANIIGGKYYGNGVTCGKHSCS VDWGKATTCIINNGAMAWATGGHQGNHKC	62	6643.64	9.02	19.72	55.16	−0.482	30 h
16	Plantaricin_W_alpha	D2KR94	MKISKIEAQARKDFFKKIDTNSNLLNVNGAKCK WWNISCDLGNNGHVCTLSHECQVSCN	59	6656.62	8.71	30.34	72.71	−0.473	30 h
17	Plantaricin_W_beta	Q9AF68	MTKTSRRKNAIANYLEPVDEKSINESFGAGDPEAR SGIPCTIGAAVAASIAVCPTTKCSKRCGKRKK	67	7085.18	9.64	47.23	61.34	−0.499	30 h
18	Sakacin_A	P0A310	MNNVKELSMTELQTITGGARSYGNGVYCNNKKCW VNRGEATQSIIGGMISGWASGLAGM	59	6257.13	8.77	29.28	67.80	−0.214	30 h
19	Sakacin_P	P35618	MEKFIELSLKEVTAITGGKYYGNGVHCGKHSCTVD WGTAIGNIGNNAAANWATGGNAGWNK	61	6385.13	7.81	21.43	64.10	−0.307	30 h
20	Melittin	DRAVPe00837	GIGAVLKVLTTGLPALISWIKRKRQQ	26	2847.49	12.02	44.73	135.00	0.273	30 h
21	UL54 (1221–1242)	DRAVPe01393	LPRRLHLEPAFLPYSVKAHECC	22	2580.06	8.06	81.98	93.18	−0.109	5.5 h

Abbreviation: GRAVY, grand average of hydropathicity.

^a^ Mammalian reticulocytes, in vitro.

The physicochemical properties of the control peptides were also less favorable in the context of MPDP targeting. Melittin had a relatively small molecular weight (2847.49 Da), a highly basic pI (12.02), a high instability index (44.73), a markedly elevated aliphatic index (135.00), and a positive GRAVY value (0.273), consistent with its strongly cationic and hydrophobic character. These features are compatible with its well‐documented broad antiviral behavior, which has frequently been linked to membrane‐associated mechanisms, but they did not correspond to superior predicted interaction with MPDP in the present study. UL54 (1221–1242) was likewise small (2580.06 Da) and moderately basic (pI 8.06), but it displayed the highest instability index among all peptides (81.98), together with a shorter estimated half‐life in mammalian reticulocytes (5.5 h). Although UL54 (1221–1242) remains a valuable mechanistic comparator because it is derived from a viral DNA polymerase and has experimentally validated polymerase‐related antiviral activity, it possessed weaker binding affinity and less favorable stability profile compared to the lead bacteriocins against MPDP.

Structural quality parameters (Table 1) indicated that both Sakacin P and Mundticin KS exhibited a high‐confidence model with an average pLDDT of 75.88 and 79.92, an LG score of 9.050 and 7.838, and 100% and 98.8% of residues in favored or allowed Ramachandran regions, respectively. Although Melittin and UL54 (1221–1242) also showed excellent model quality, their lower docking affinities and less favorable physicochemical properties relative to Sakacin P and Mundticin KS indicated that structural validity alone was insufficient to determine lead selection. Rather, Sakacin P and Mundticin KS were distinguished by the most favorable overall balance of predicted MPDP binding and physicochemical suitability.

The structural validations of the Sakacin P and Mundticin KS were illustrated in Figures S1 and S2. Their Ramachandran plot showed most residues fall within favored or allowed regions, indicating good backbone geometry and the predicted secondary structure and solvent accessibility of the peptide were visualized (Figures S1B,C and S2B,C). The map of each residues to its Ramachandran classification confirmed that the majority are in favored or allowed regions (Figures S1D and S2D). Overall, the models for both bacteriocins demonstrated good structural quality with minimal stereochemical outliers.

**FIGURE 2:**
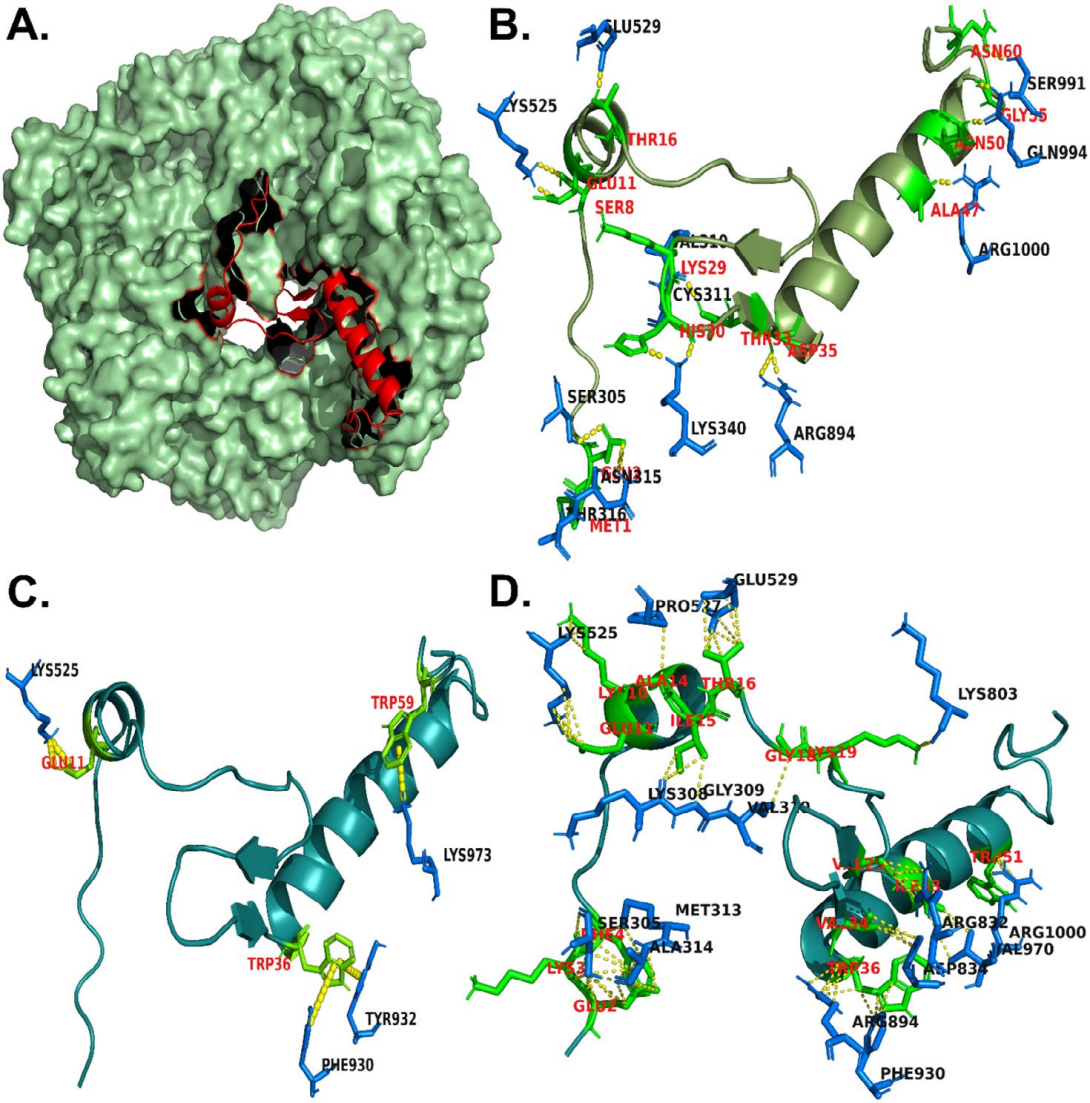
(A) Surface representation of MPDP showing the DNA‐binding groove (green) with Sakacin P (red cartoon) docked within the predicted binding pocket. (B) Hydrogen‐bond interactions between Sakacin P and MPDP. Residues of MPDP involved in hydrogen bonding are highlighted in blue, whereas interacting residues of Sakacin P are shown in green. (C) Non‐covalent interactions identified using the RING 4.0 server, including an ionic interaction between LYS‐525 (Chain A) and GLU‐11 (Chain B), a π–cation interaction between LYS‐973 (Chain A) and TRP‐59 (Chain B), and π–π stacking interactions involving PHE‐930 (Chain A), TYR‐932 (Chain A), and TRP‐36 (Chain B). MPDP residues are shown in blue, whereas interacting residues of Sakacin P are shown in green. (D) Van der Waals contacts between residues of the MPDP DNA‐binding groove (blue) and interacting residues of Sakacin P (green). The interacting residue labels belonging to Sakacin P were displayed as red, whereas the interacting residue labels from MPDP were shown as black.

The surface illustration of the DNA‐binding groove of MPDP and an insertion view of Sakacin P into the DNA‐binding site of MPDP were also shown in Figure 2A. A comprehensive residue interaction analysis was performed using the RING 4.0 tool, which revealed an extensive network of non‐covalent contacts between Sakacin P and MPDP. The interactions, including hydrogen bonds, π–π stacking, π–cation interactions, and van der Waals forces, are expected to contribute to the stability and specificity of the complex. The interactions associated with the docking pose of Sakacin P are summarized in Table 3. The hydrogen bonds identified for Sakacin P included A305‐B2, A310‐B33, A311‐B29, A315‐B2, A316‐B1, A340‐B30, B525‐B8, A525‐B11, A529‐B16, A894‐B35, A991‐B60, A994‐B50, A994‐B55, and A1000‐B47 (Figure 2B). The hydrogen bonds among the active residues of MPDP and Sakacin P at A340‐B30, B525‐B8, A525‐B11, and A1000‐B47 were readily observable, contributing receptor–ligand binding stability. Hydrogen bonding is a weak electrostatic attraction that occurs between a hydrogen atom covalently bonded to a highly electronegative atom (such as oxygen, nitrogen, or fluorine), and another electronegative atom in a separate molecule or within a different region of the same molecule (Torshin et al. 2002).

**TABLE 3:** List of interchain interactions that exist between monkeypox virus DNA polymerase and Sakacin P.

Hydrogen bonds		Ionic interactions		Van der Waals forces	
Chain A (8HOY)	Chain B (Sakacin P)	Chain A (8HOY)	Chain B (Sakacin P)	Chain A (8HOY)	Chain B (Sakacin P)
305‐Ser	2‐Glu	**525‐Lys**	11‐Glu	305‐Ser	2‐Glu
310‐Val	33‐Thr	**π–cation**		305‐Ser	3‐Lys
311‐Cys	29‐Lys	**973‐Lys**	59‐Trp	**308‐Lys**	15‐Ile
315‐Asn	2‐Glu	**π–π stack**		309‐Gly	15‐Ile
316‐Thr	1‐Met	930‐Phe	36‐Trp	310‐Val	18‐Gly
**340‐Lys**	30‐His	932‐Tyr	36‐Trp	313‐Met	4‐Phe
**525‐Lys**	8‐Ser			314‐Ala	2‐Glu
**525‐Lys**	11‐Glu			**525‐Lys**	10‐Lys
529‐Glu	16‐Thr			**525‐Lys**	11‐Glu
894‐Arg	35‐Asp			527‐Pro	14‐Ala
991‐Ser	60‐Asn			529‐Glu	16‐Thr
994‐Gln	50‐Asn			**803‐Lys**	19‐Lys
994‐Gln	55‐Gly			**832‐Arg**	25‐Val
**1000‐Arg**	47‐Ala			834‐Asp	34‐Val
				894‐Arg	36‐Trp
				930‐Phe	36‐Trp
				970‐Val	43‐Ile
				**1000‐Arg**	51‐Trp

*Note:* The amino acids highlighted in bold represent the active residues of MPDP.

The docking pose of Sakacin P revealed the presence of an ionic interaction between A525‐B11, a π–cation bond between A973‐B59, and π–π stacking interactions between A930‐B36 and A932‐B36 in conjunction with MPDP and Sakacin P (Figure 2C). In addition to hydrogen bonds, there were other non‐covalent interactions that contribute to receptor‐ligand interaction. Ionic interactions arise from electrostatic attraction between oppositely charged groups, such as positively charged amino groups and negatively charged carboxylate groups present in amino acid side chains (Del Conte et al. 2024). The π–π stacking refers to non‐covalent interactions between aromatic residues through favorable alignment of electron‐rich π orbitals, which can enhance structural stabilization through attractive intermolecular forces (Zhao et al. 2015). Similarly, π–cation interactions occur between a positively charged group and the electron‐rich π system of an aromatic group. The π–cation interactions are known to contribute to protein structural stability, conformational regulation, and ligand recognition. Interactions involving basic and aromatic amino acids are particularly important in membrane proteins, where they can help stabilize the overall structure (Infield et al. 2021). In the Sakacin P docking pose, the observed van der Waals forces were as follows: A305‐B2, A305‐B3, A308‐B15, A309‐B15, A310‐B18, A313‐B4, A314‐B2, A525‐B10, A525‐B11, A527‐B14, A529‐B16, A803‐B19, A832‐B25, A834‐B34, A894‐B36, A930‐B36, A970‐B43, and A1000‐B51. Particularly, van der Waals forces between MPDP active residues and Sakacin P (A308‐B15, A525‐B10, A525‐B11, A803‐B19, A832‐B25, and A1000‐B51) contributed significantly to the inhibitory effect of Sakacin P (Figure 2D). Additionally, van der Waals forces facilitated close geometric complementarity between the protein and bacteriocin by improving local atomic packing, particularly in hydrophobic regions of the interface. These detailed interactions underlined the synergistic effects of multiple non‐covalent forces in stabilizing the predicted complex (van Oss et al. 1980, 1986; Varma et al. 2010).

**FIGURE 3:**
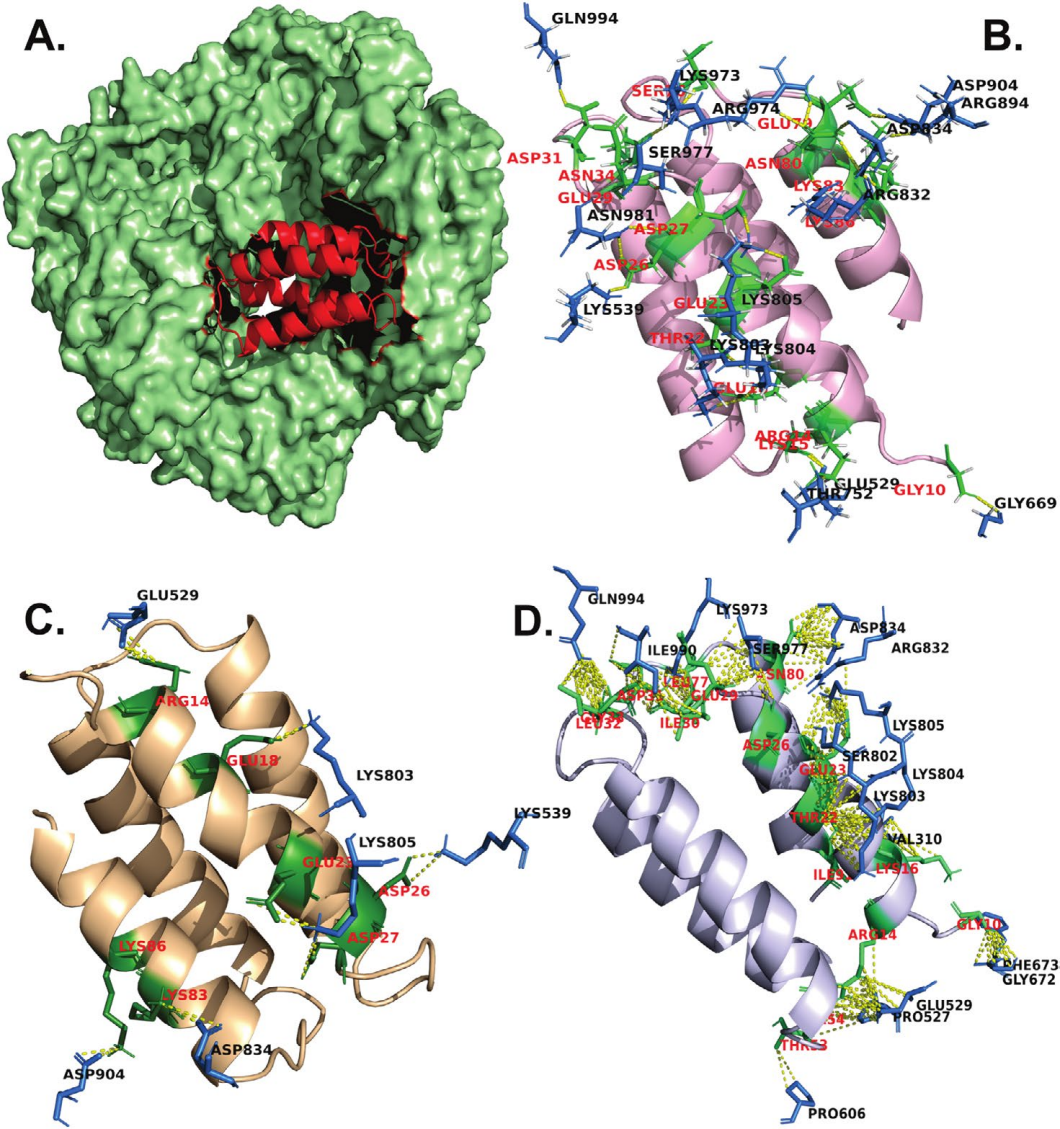
(A) Surface representation of MPDP showing the DNA‐binding groove (green) with Mundticin KS (red cartoon) docked within the predicted binding pocket. (B) Hydrogen‐bond interactions between Mundticin KS and MPDP. Residues of MPDP involved in hydrogen bonding are highlighted in blue, whereas interacting residues of Mundticin KS are shown in green. (C) Ionic interactions between the DNA‐binding groove of MPDP (Chain A) and Mundticin KS (Chain B), as identified using the RING 4.0 server. MPDP residues participating in ionic contacts are shown in blue, while interacting Mundticin KS residues are shown in dark green. (D) Van der Waals contacts between residues of the MPDP DNA‐binding groove (blue) and interacting residues of Mundticin KS (green). The interacting residue labels for Mundticin KS were displayed in red, whereas those from MPDP were shown in black.

The docking pose of Mundticin KS revealed a dense network of non‐covalent interactions with MPDP, including multiple hydrogen bonds, ionic interactions, and van der Waals contacts (Figure 3A and Table 4). Hydrogen bonding interactions were observed between residues of MPDP and Mundticin KS, notably involving A529‐B14, A539‐B26, A669‐B10, A752‐B15, A803‐B18, A804‐B18, A804‐B22, A805‐B23, A805‐B27, A832‐B80, A834‐B80/b83, A894‐B79, A904‐B86, A973‐B34/B76, A974‐B80, A977‐B27/B29, A981‐B26, and A994‐B31 (Figure 3B). These interactions indicate extensive polar contact formation between Mundticin KS and residues distributed across the MPDP binding interface. In addition, ionic interactions were identified between several charged residue pairs, including A529‐B14, A539‐B26, A803‐B18, A805‐B23/B27, A834‐B83, and A904‐B86, highlighting strong electrostatic complementarity between the Mundticin KS and MPDP (Figure 3C). Notably, several interactions involved the active residues of MPDP, contributing receptor‐ligand binding stability. Van der Waals forces were widely distributed across the interface of Mundticin KS and MPDP (Figure 3D). These contacts indicate extensive shape complementarity and close packing between MPDP and Mundticin KS, particularly around the DNA‐binding groove of MPDP. Collectively, these synergistic interactions supports the formation of a stable protein–peptide complex and can suggest that Mundticin KS may effectively engage the MPDP binding interface.

**TABLE 4:** List of interchain interactions exist monkeypox virus DNA polymerase and Mundticin KS.

Hydrogen bonds		Ionic interactions		Van der Waals forces	
Chain A (8HOY)	Chain B (Mundticin KS)	Chain A (8HOY)	Chain B (Mundticin KS)	Chain A (8HOY)	Chain B (Mundticin KS)
529‐Glu	14‐Arg	529‐Glu	14‐Arg	310‐Val	16‐Lys
539‐Lys	26‐Asp	539‐Lys	26‐Asp	310‐Val	91‐Ile
669‐Gly	10‐Gly	**803‐Lys**	18‐Glu	527‐Pro	54‐Ser
752‐Thr	15‐Lys	**805‐Lys**	23‐Glu	529‐Glu	14‐Arg
803‐Lys	18‐Glu	**805‐Lys**	27‐Asp	606‐Pro	53‐Thr
804‐Lys	18‐Glu	834‐Asp	83‐Lys	672‐Gly	10‐Gly
804‐Lys	22‐Thr	904‐Asp	86‐Lys	673‐Phe	10‐Gly
**805‐Lys**	23‐Glu			802‐Ser	22‐Thr
**805‐Lys**	27‐Asp			**803‐Lys**	22‐Thr
**832‐Arg**	80‐Asn			**804‐Lys**	22‐Thr
834‐Asp	80‐Asn			**805‐Lys**	23‐Glu
834‐Asp	83‐Lys			832‐Arg	23‐Glu
894‐Arg	79‐Glu			834‐Asp	80‐Asn
904‐Asp	86‐Lys			**973‐Lys**	77‐Leu
**973‐Lys**	34‐Asn			977‐Ser	26‐Asp
**973‐Lys**	76‐Ser			977‐Ser	29‐Glu
**974‐Arg**	80‐Asn			990‐Ile	30‐Ile
977‐Ser	27‐Asp			990‐Ile	31‐Asp
977‐Ser	29‐Glu			994‐Gln	32‐Leu
981‐Asn	26‐Asp			994‐Gln	33‐Gly
994‐Gln	31‐Asp			**1000‐Arg**	31‐Asp

*Note:* The amino acids highlighted in bold represent the active residues of MPDP.

Overall, the superior binding affinity and balanced physicochemical characteristics of Sakacin P and Mundticin KS highlighted their potential as candidate inhibitors of MPDP. Future studies should focus on in vitro and in vivo validation, as well as chemical modifications to enhance selectivity and bioavailability of these bacteriocins.

### 3.3 Molecular Dynamics Simulations

**FIGURE 4:**
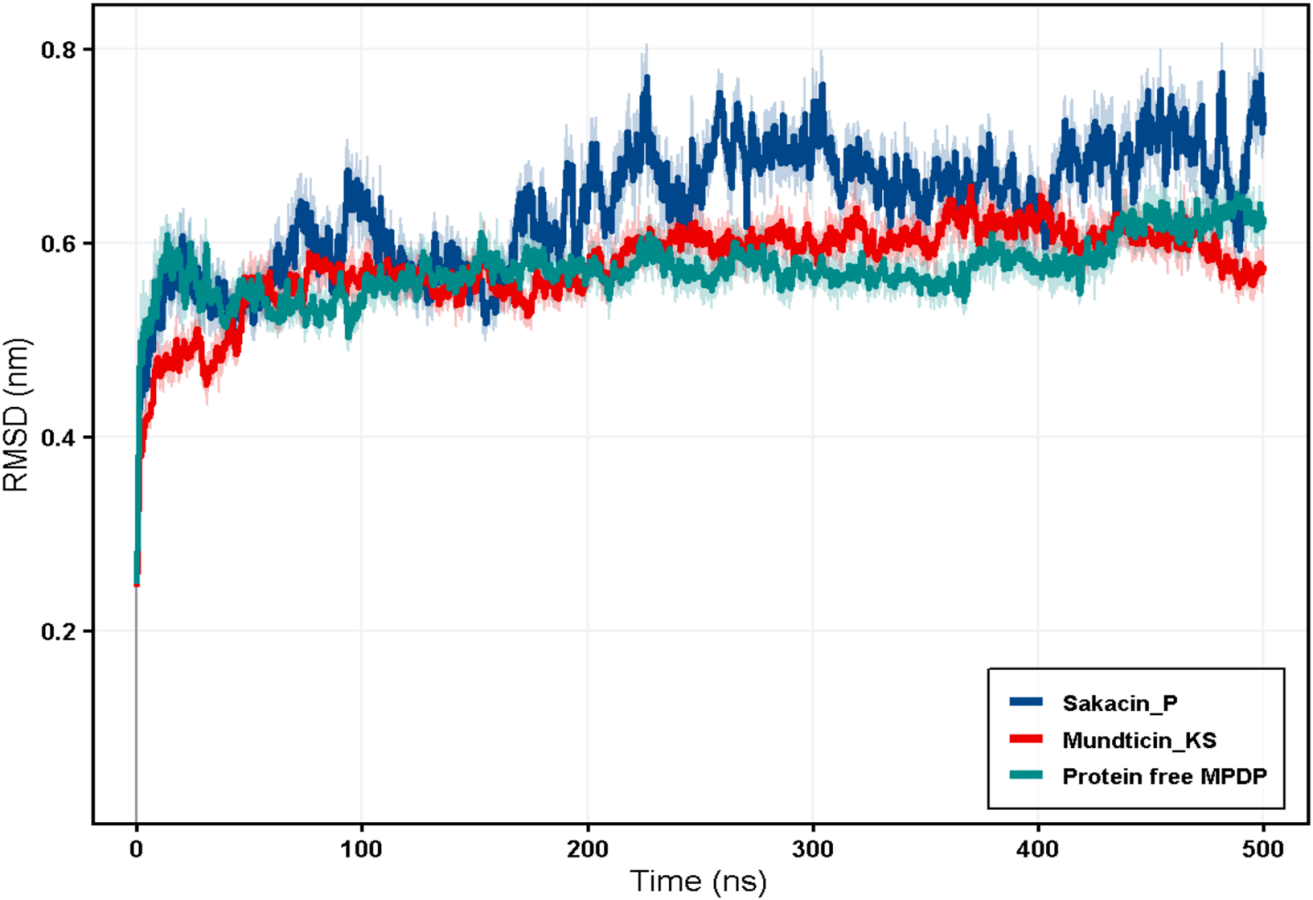
Root mean square deviation (RMSD) profiles of MPDP in its unbound (free) form and in complex with Sakacin P and Mundticin KS during the molecular dynamics simulation. RMSD values were calculated over the simulation time to evaluate structural stability and conformational changes upon peptide binding.

Sakacin P and Mundticin KS complexed with MPDP were further analyzed via molecular dynamics simulations to validate docking results. MD simulations were conducted with GROMACS for 500 ns. Firstly, Figure 4 shows the comparisons of root mean square deviations (RMSD) for two states of a protein: protein complexed with bacteriocins (blue and red) and protein free (green). Both states started with low RMSD values (~0.4–0.5 nm), indicating minimal deviation from their respective initial structures. Then, a sharp increase was observed at the beginning, likely representing the system moving out of the energy‐minimized starting configuration to explore conformational space. After that, RMSD stabilized relatively quickly around ~0.5–0.6 nm for free protein, suggesting it was equilibrated. For the complexed protein with Sakacin P, RMSD stabilized later (~200–300 ns) and at a higher average (~0.6–0.7 nm). This may reflect conformational changes or flexibility due to interactions with the binding peptide. In addition, the complexed protein with Sakacin P showed higher fluctuations compared to the free protein throughout the simulation. This may indicate the dynamic behavior induced by binding interactions and a larger conformational search space due to the influence of the Sakacin P. On the other hand, RMSD stabilized relatively quickly around ~0.5–0.6 nm for complexed protein with Mundticin KS, suggesting it was equilibrated. Overall, stable RMSDs after initial equilibration were observed for all runs, implying no significant structural destabilization during the simulated time. Particularly, complexed protein with Mundticin KS showed similar RMSD profile to the free protein. Mundticin KS confered greater stability than Sakacin P, and at several points, it is even more stable than the free protein (Figure 4).

**FIGURE 5:**
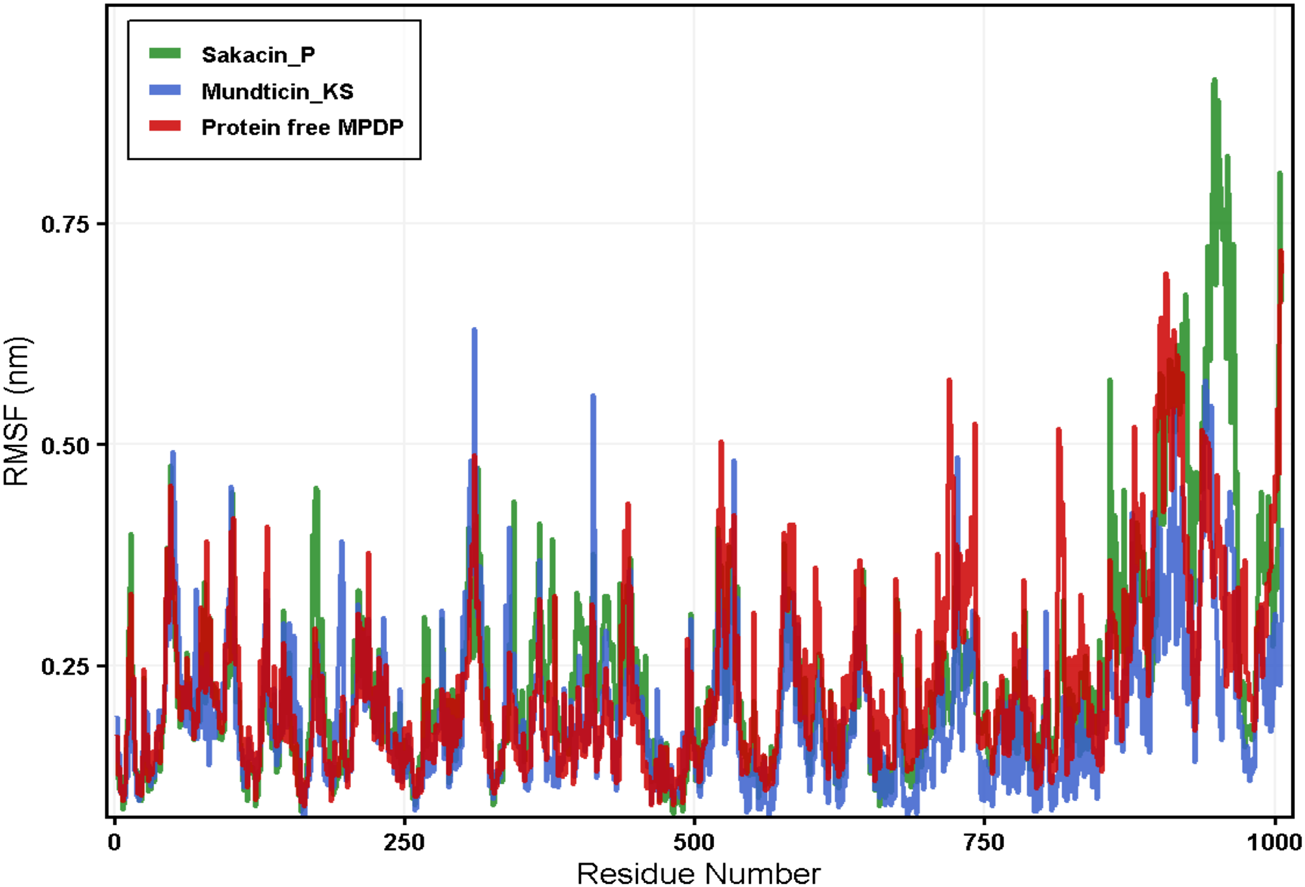
Root mean square fluctuation (RMSF) profiles of MPDP residues in the unbound (free) state and in complex with Sakacin P and Mundticin KS during the molecular dynamics simulation. RMSF values were calculated to evaluate residue‐level flexibility and to identify regions affected by peptide binding.

Furthermore, root mean square fluctuation (RMSF) analysis for the protein‐complexed with Sakacin P and Mundticin KS and free protein were conducted (Figure 5). RMSF is a measure of the flexibility of each residue over the simulation time, with higher values indicating more flexibility. All three systems exhibited distinct regions of higher and lower RMSF, suggesting that some residues are inherently more flexible than others, regardless of the state. The RMSF values were generally higher for the free protein, indicating that it is more flexible overall compared to the complexed proteins. The Sakacin P and Mundticin KS likely stabilized the protein structure, reducing the fluctuations, especially in regions directly or indirectly interacting with the ligand. Among the two complexes, the protein complexed with Mundticin KS system displayed lower RMSF values than the Sakacin P complex across several residue segments, suggesting comparatively stronger local stabilization. The peaks of fluctuation were observed in loop regions or termini, which are typically less structured and more flexible. Higher RMSF peaks in the free state suggested that ligand binding reduced flexibility in some regions, possibly locking them into more rigid conformations. Near the C‐terminal region (approximately residue index 1000), both the free protein and the protein complexed with Sakacin P exhibited increased fluctuations, whereas the MPDP‐Mundticin KS complex maintained comparatively moderate RMSF values without a sharp increase. This observation may indicate enhanced stabilization of terminal regions by Mundticin KS. Overall, the complexed proteins indicated reduced flexibility due to stabilization from interactions with the bacteriocins. This is typical for ligand‐binding proteins, where binding induces a conformational change or rigidity in certain regions.

**FIGURE 6:**
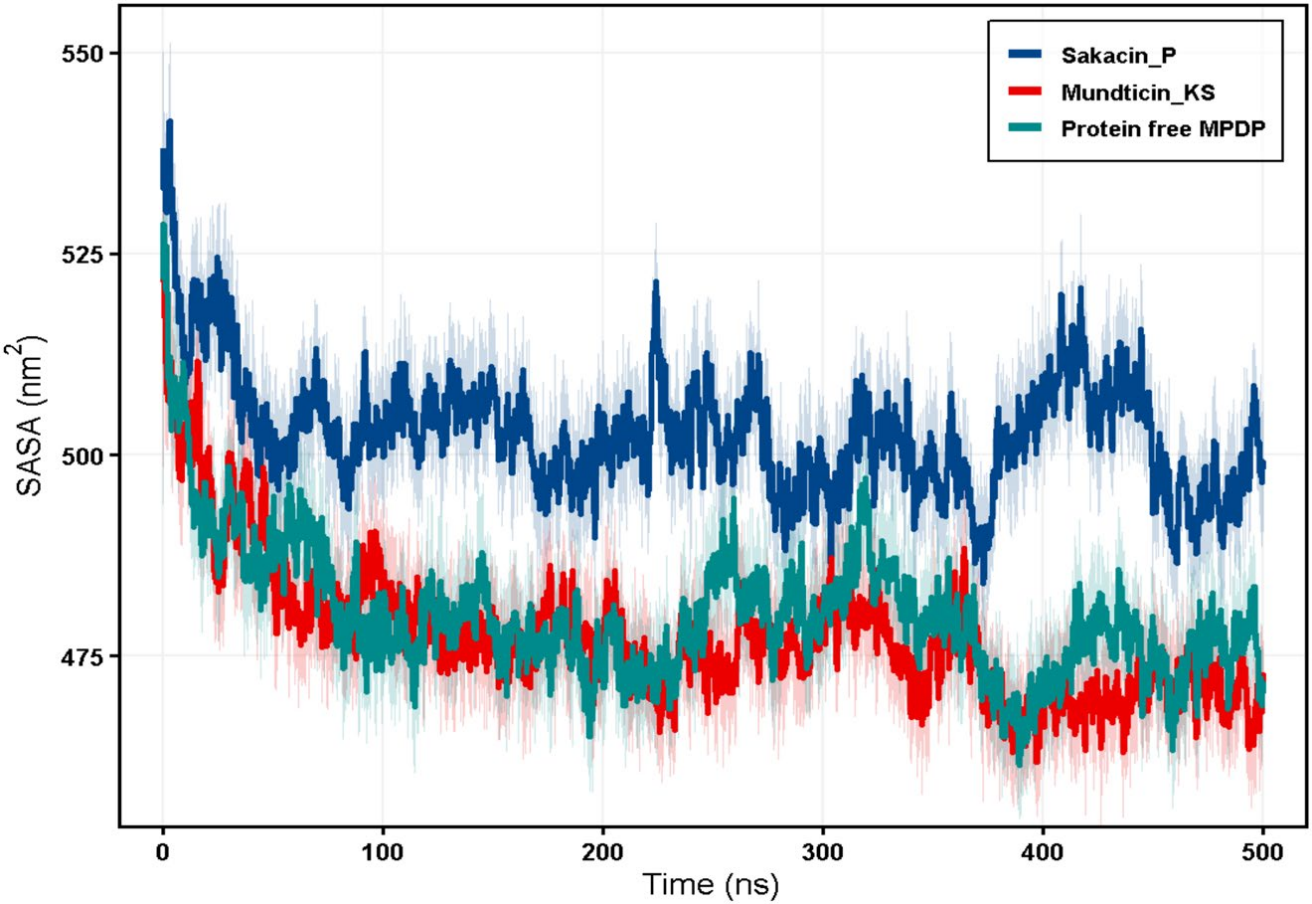
Solvent‐accessible surface area (SASA) of MPDP in its unbound (free) state and in complex with Sakacin P and Mundticin KS during the molecular dynamics simulation. The analysis reflects changes in the exposure of the protein surface and provides insight into conformational rearrangements induced by peptide binding.

**FIGURE 7:**
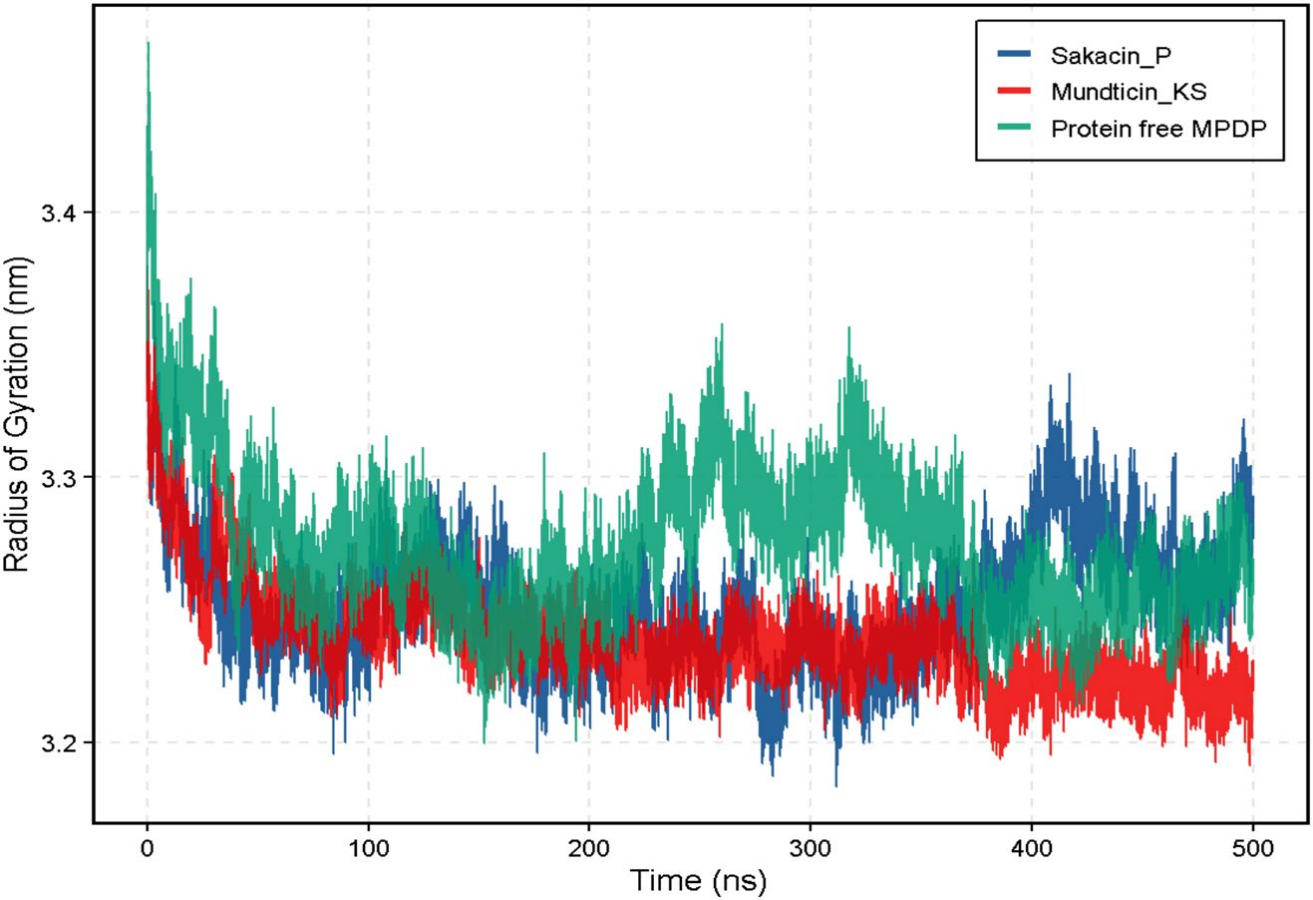
Radius of gyration (Rg) profiles of MPDP in its unbound (free) state and in complex with Sakacin P and Mundticin KS during the molecular dynamics simulation. Rg values were calculated over the simulation time to assess the overall compactness and structural stability of the protein upon peptide binding.

Figure 6 shows the solvent‐accessible surface area (SASA) of three complex systems: free protein and protein complexed with Sakacin P and Mundticin KS. All three systems exhibited relatively stable SASA values over time, indicating minimal structural fluctuations. However, the complexed protein with Sakacin P had consistently higher SASA values than the free protein, suggesting that binding to Sakacin P may expose more surface area to the solvent, whereas the complexed protein with Mundticin KS had similar SASA values to the free protein. After the equilibration, SASA stabilized around 490–520 nm^2^ for the complexed protein with Sakacin P with some fluctuations. The binding event in the complexed protein with Sakacin P may increase conformational flexibility, leading to a more solvent‐exposed structure. It is also indicative of the stabilization of the structure, which could prevent compaction, maintaining a higher SASA. For the complexed protein with Mundticin KS, SASA stabilized around 465–485 nm^2^ in a similar manner to the free protein with relatively less fluctuations. The similar SASA profile might indicate that Mundticin KS did not lead to conformational rearrangements induced by peptide binding. Figure 7 represents the radius of gyration (Rg) over time for three complex systems. Both the free protein and protein complexed with bacteriocins started with a relatively high Rg value (~3.45–3.50 nm). A sharp drop in Rg occurred within the first ~50 ns, which indicates an initial compaction of all structures. The drop was more significant in the protein complexed with bacteriocins, suggesting the complexation induces a tighter conformation. After ~100 ns, all structures stabilized. The protein complexed with Sakacin P form had a lower Rg compared to free protein, indicating that the bound state maintained a more compact structure. Beyond 300 ns, the Rg values for both free protein and protein complexed with Sakacin P appeared to fluctuate within a stable range. The protein complexed with Sakacin P remained consistently lower (~3.25–3.3 nm), while the free protein fluctuated around 3.25–3.35 nm. This supported the idea that the binding of Sakacin P stabilized the protein structure, reducing overall flexibility. On the other hand, after initial compaction, protein complexed with Mundticin KS had lower Rg values than free protein and protein complexed with Sakacin P, and remained stable throughout the simulation period with a Rg value of 3.20–3.25 nm, indicating overall compactness and structural stability of the protein upon peptide binding.

**FIGURE 8:**
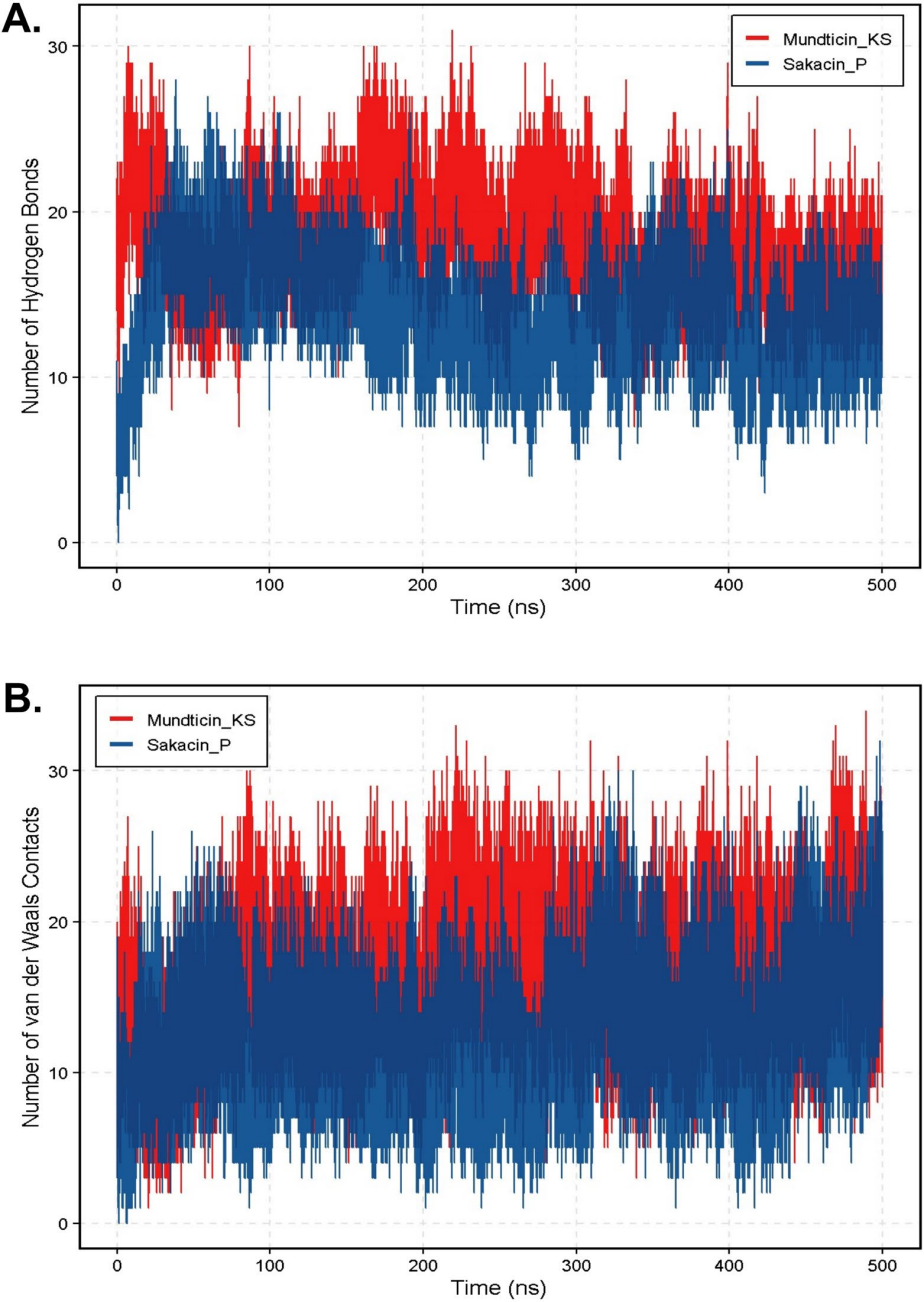
Analysis of intermolecular interactions between MPDP and bacteriocins (Sakacin P and Mundticin KS) during molecular dynamics simulations: (A) number of hydrogen bonds and (B) van der Waals interaction energy. These parameters were monitored over the simulation time to evaluate the stability and strength of protein–peptide interactions.

The number of hydrogen bonds (H‐bonds) and van der Waals (vdW) interactions formed between MPDP and bacteriocins throughout the 500 ns molecular dynamics (MD) simulation were demonstrated in Figure 8. The number of hydrogen bonds quickly fluctuated and stabilized around 8–20 bonds for the protein complexed with Sakacin P. This suggested that the complex quickly established consistent intermolecular interactions after the simulation begins. This indicated a stable binding interface, implying that the protein–ligand complex was well‐formed and hold together by persistent H‐bond interactions. For the protein complexed with Mundticin KS, the number of hydrogen bonds quickly fluctuated and stabilized around 10–30 bonds, indicating stronger stability compared to Sakacin P. Overall, the H‐bonding profile reflected a dynamic but generally stable interaction between the protein and bacteriocins for the majority of the simulation. Throughout most of the simulations, vdW interactions fluctuated between 5 and 30 contacts for both complexes, indicating a persistent and strong non‐covalent interaction between the protein and ligands. The frequency and density of these interactions suggested that both bacteriocins remained closely associated with the protein, contributing to the stability of the complex. Despite the short‐lived drops, the system consistently reestablished strong vdW interactions, indicating robust binding and favorable surface complementarity between the interacting partners. No long‐term loss of interactions was observed for both complexes, supporting the structural and energetic stability of the complex. The dominance of vdW interactions, together with hydrogen bonding data, supported the stable and possibly functionally relevant association of bacteriocins within the binding site of MPDP. Particularly, Munditicin KS had higher number of H‐bond and vdW interactions, showing stronger affinity and stability within the binding site of MPDP.

The ligand–receptor interaction analysis was further performed using MM‐PBSA binding energy calculations. The contributions of enthalpy and entropy changes for MPDP complexed with Sakacin P were found as −182.12 and 68.93 kcal/mol, respectively. This resulted in a Gibbs free binding energy change of −113.19 kcal/mol. The contributions of enthalpy and entropy changes for MPDP complexed with Mundticin KS were found as −146.61 and 18.55 kcal/mol, respectively, yielding a Gibbs free binding energy of −128.06 kcal/mol. The more negative Gibbs free binding energy change value suggests a stronger predicted binding affinity of Mundticin KS toward MPDP compared with Sakacin P. indicating a stronger binding affinity between Mundticin KS and MPDP compared to the one with Sakacin P. Collectively, these findings indicate that both peptide–protein complexes remained stable during the 500 ns molecular dynamics simulations, thereby supporting the molecular docking results and the potential of both peptides as candidate MPDP inhibitors. Based on the MM‐PBSA energy estimates and dynamic behavior observed during simulation, Mundticin KS may represent the more favorable candidate, whereas Sakacin P also demonstrated stable and energetically favorable binding.

## 4 Discussion

In this study, bacteriocins from diverse origins were virtually screened against the MPDP using the HADDOCK interface. Among the bacteriocins tested, Mundticin KS, Sakacin P, and Plantaricin W beta showed the highest binding affinities for the MPDP with Δ*G* values of −17.0, −16.7, and −16.2 kcal/mol, respectively. Physicochemical analysis showed that Mundticin KS and Plantaricin W beta had higher instability index scores exceeding the threshold value of 42, whereas Sakacin P demonstrated good stability with a low instability index score. Based on these findings, Sakacin P and Mundticin KS were selected for MD simulation to evaluate their stability and dynamics in the context of the DNA replication complex. The 500 ns MD simulations showed the stabilities of Sakacin P and Mundticin KS when bound to MPDP, indicating their strong potential as MPDP inhibitor candidates for further experimental investigation.

Plantaricin W is synthesized by *Lactobacillus plantarum* , consisting of two complementary peptides that act synergistically to exhibit antimicrobial effects against a broad range of Gram‐positive bacteria, including several foodborne pathogens. It also showed a remarkable anti‐Listeria activity (Holo et al. 2001; Tenea and Pozo 2019; Wiman et al. 2023). Furthermore, Plantaricin W also exhibited heat stability and maintained its activity over a wide pH range, supporting its potential application in food preservation. The antifungal property of Plantaricin was demonstrated against a diverse range of fungal species, including *Penicillium roqueforti*, *Mucor plumbeus*, *Penicillium expansum*, *Cladosporium* sp., *Fusarium* sp., and *Debaromyces hansenii* (Barbosa et al. 2016). Computational in silico research showed that Plantaricin W might have antiviral properties against COVID‐19 by strongly binding to the main druggable targets of SARS‐CoV‐2 (Anwar et al. 2021). Similarly, Plantaricin NC8 αβ was found to rapidly and effectively inhibit flaviviruses and SARS‐CoV‐2 by disrupting their viral envelopes (Omer et al. 2022).

Mundticin KS is a bacteriocin produced by *Enterococcus mundtii* , a species that may occur as a commensal organism in the intestinal tract and is also frequently isolated from fermented foods. This strain boasts antimicrobial activity primarily against a relatively narrow range of related lactic acid bacteria and the foodborne pathogen *Listeria monocytogenes* . The characterization of the purified peptide demonstrated that Mundticin KS was a small cationic peptide with remarkable heat stability, retaining significant antimicrobial activity even after exposure to temperatures up to 100°C. It also remains stable over a broad pH range. In addition, the peptide is sensitive to trypsin and proteinase K, but resistant to lipase and RNase A (Kawamoto et al. 2002). Mundticin KS, produced by *E. mundtii* Tw56, was also reported to exhibit a broad spectrum of activity, effectively targeting *Pseudomonas aeruginosa* and *Shewanella putrefaciens* (Schelegueda et al. 2015). While a bacteriocin synthesized by *E. mundtii* ST4V demonstrated broad antiviral activity against Herpes simplex viruses HSV‐1 and HSV‐2, Polio virus, and Measles virus (Todorov et al. 2010), no antiviral activity has been specifically reported for Mundticin KS.

Sakacin P is a lantibiotic antimicrobial peptide naturally produced during the fermentation by *L. sakei* , which is a common bacterium in various fermented foods (Mathiesen et al. 2005; Drider et al. 2006). It is a small, positively charged peptide with antimicrobial activity and a relatively narrow target spectrum. Although it shows limited antibacterial effect on Gram‐negative bacteria, it strongly inhibits the growth of the pathogenic *Listeria monocytogenes* , a major cause of foodborne illness (Chang et al. 2022). Additionally, both Sakacin P and the producer strain *L. sakei* have been shown to possess capabilities in controlling *L. monocytogenes* contamination in chicken cold cuts (Katla et al. 2002). Furthermore, Sakacin P‐producing *L. sakei* showed preventive effects against carbapenem‐resistant *Klebsiella pneumoniae* (CRKP) infections, with lyophilized probiotics reducing body weight loss, mortality, and disease severity in CRKP‐infected mice (Tajdozian et al. 2024). Likewise, *L. sakei* HEM 224 isolated from traditional Korean kimchi was shown to alleviate inflammation in the gastrointestinal and respiratory tracts through increased epithelial barrier function and immune modulation (Kim et al. 2023). Additional studies reported that probiotic *L. sakei* significantly suppressed inflammatory responses in an animal model of rheumatoid arthritis by modulating differentiation of Th17 and regulatory B cells and blocking osteoclast formation (Jhun et al. 2020). Furthermore, the probiotic *L. sakei* Pro Bio65 was found to reduce TNFα levels, to increase glutathione concentrations, and to stimulate antioxidant enzyme activities in human conjunctival cells, while the extract from this probiotic also showed potent antiviral effects against SARS‐CoV‐2 infection in vitro (Iorio et al. 2021; Rather et al. 2022). Notably, Sakacin P was found to have a strong binding affinity for the human ACE2 receptor in an in silico study, which implied its potential antiviral activity against SARS‐CoV‐2 (Manna et al. 2021). These investigations have convincingly revealed that *L. sakei* along with its bacteriocin Sakacin P has a considerable therapeutic potential for the treatment of different diseases. Although a few studies have explored the antiviral potential of Sakacin P against SARS‐CoV‐2 infection, to the best of our knowledge no previous study has investigated its possible antiviral activity against MPXV. Hence, the present study offers the first evidence on the potential antiviral properties of Sakacin P against MPXV.

## 5 Conclusion

This study reveals the promising nature of bacteriocins to act as MPDP inhibitors using computational methods. Structural modeling, molecular docking, and MD simulations collectively indicated that Sakacin P and Mundticin KS exhibited superior binding affinities and stabilities in complex with MPDP. The presence of essential bonding interactions, such as hydrogen bonds, ionic interactions, and π–π stacking, was a major factor in the ligand binding stability. The conformational stabilities of the protein–ligand complexes were verified through MD simulations. These results represent a significant move toward the rational design of bacteriocin‐based antiviral agents against MPXV. Future studies should focus on experimental validation of these interactions to explore the therapeutic potential of bacteriocins in combating MPXV infections. The antiviral potential of Sakacin P and Mundticin KS needs to be confirmed by further in vitro and in vivo studies.

## Author Contributions

**Melisa Z. Karaman:** investigation, writing – original draft, writing – review and editing, visualization, methodology, formal analysis, data curation. **Karan Goel:** writing – review and editing. **Fernando Berton Zanchi:** methodology, validation, formal analysis, data curation, software. **Aykut Ozdarendeli:** writing – review and editing, methodology, conceptualization. **Somdutt Mujwar:** writing – review and editing. **Ahmet E. Yetiman:** conceptualization, investigation, methodology, visualization, validation, writing – review and editing, writing – original draft, project administration, data curation, formal analysis, supervision, resources. **Ozkan Fidan:** conceptualization, investigation, writing – original draft, writing – review and editing, project administration, supervision, resources.

## Conflicts of Interest

The authors declare no conflicts of interest.

## Supporting information


**Figure S1:** Three‐dimensional structure of Sakacin P (P35618) from *Latilactobacillus sakei* and its confirmation via Ramachandran plot. (A) Cartoon illustration of 3D structure of Sakacin P. (B) Verification of the 3D structure predicted via the Ramachandran plot. (C) Description of the secondary structure of Sakacin P, which is composed of α‐helices, β‐sheets, and random‐coiled polypeptide structures. (D) The peptide sequence and Ramachandran regions are illustrated.
**Figure S2:** Three‐dimensional structure of Mundticin KS (2ZRR) from *Enterococcus mundtii* and its confirmation via Ramachandran plot. (A) Cartoon illustration of 3D structure of Mundticin KS. (B) Verification of the 3D structure predicted via the Ramachandran plot. For Mundticin KS, 93.4% of residues (black dots) have been determined in the favored areas. (C) Description of the secondary structure of Mundticin KS, which is composed of α‐helices and random‐coiled polypeptide structures. (D) The peptide sequence and Ramachandran regions are illustrated.

## Data Availability

The data supporting the findings of this study are openly available in the Zenodo repository at https://doi.org/10.5281/zenodo.19671741.
