# Supplementary material for: Virtual Screening of Bacteriocins From Lactic Acid Bacteria Against Monkeypox DNA Polymerase: Sakacin P and Mundticin KS Emerge as Promising Candidates
Source: Food Sci Nutr. 2026 Aug 6;14(8):e72219. doi: 10.1002/fsn3.72219 (PMC13447968; doi:10.1002/fsn3.72219)
Supplement: Supplementary file 1 [file FSN3-14-e72219-s001.docx]

**Supplementary File for**

**Virtual Screening of Bacteriocins from Lactic Acid Bacteria against Monkeypox DNA Polymerase: Sakacin P and Mundticin KS emerge as Promising Candidates**

Melisa Z. Karaman^1^, Karan Goel^2^, Fernando Berton Zanchi^3^, Aykut Ozdarendeli ^4,5^, Somdutt Mujwar^2^, Ahmet E. Yetiman^6, *^, Ozkan Fidan^1, *^

^1^ Department of Bioengineering, Faculty of Life and Natural Sciences, Abdullah Gul University, Kayseri, Turkiye, 38080

^2^ Chitkara College of Pharmacy, Chitkara University, Rajpura-140401, Punjab, India

^3^ Bioinformatic and Medicinal Chemistry Laboratory, Fiocruz/RO, Porto Velho, Rondônia, Brazil, 76812-245

^4^ Department of Microbiology, Faculty of Medicine, Erciyes University Kayseri, Türkiye, 38280

^5^ Vaccine Research, Development and Application Centre (ERAGEM), Erciyes University, Kayseri, Türkiye, 38280

^6^ Department of Food Engineering, Faculty of Engineering, Erciyes University, Kayseri, Turkiye, 38280

*Corresponding authors: [ahmetyetiman@erciyes.edu.tr](mailto:ahmetyetiman@erciyes.edu.tr) and [ozkan.fidan@agu.edu.tr](mailto:ozkan.fidan@agu.edu.tr)

**Fig. S1.** Three-dimensional structure of Sakacin P (P35618) from Latilactobacillus sakei and its confirmation via Ramachandran plot. (A) Cartoon illustration of 3D structure of Sakacin P. (B) Verification of the 3D structure predicted via the Ramachandran plot. (C) Description of the secondary structure of Sakacin P, which is composed of α-helices, β-sheets, and random-coiled polypeptide structures. (D) The peptide sequence and Ramachandran regions are illustrated.

**Fig. S2.** Three-dimensional structure of Mundticin KS (2ZRR) from *Enterococcus mundtii* and its confirmation via Ramachandran plot. (A) Cartoon illustration of 3D structure of Mundticin KS. (B) Verification of the 3D structure predicted via the Ramachandran plot. For Mundticin KS, 93.4% of residues (black dots) have been determined in the favored areas. (C) Description of the secondary structure of Mundticin KS, which is composed of α-helices and random-coiled polypeptide structures. (D) The peptide sequence and Ramachandran regions are illustrated.
